# Supplementary material for: Hydroxypropyl-Beta Cyclodextrin Barrier Prevents Respiratory Viral Infections: A Preclinical Study
Source: Int J Mol Sci. 2024 Feb 8;25(4):2061. doi: 10.3390/ijms25042061 (PMC10888609; doi:10.3390/ijms25042061)
Supplement: Supplementary file 1 [file ijms-25-02061-s001.zip › ijms-2784733-supplementary.pdf]

Supplementary Material

Supplementary Table S1: SARS-CoV-2 pseudotype variant classification. * SARS-CoV-2 Interagency Group (SIG) classification: Variant Being Monitored (VBM); Variant of Concern (VOC).

Pango Lineage	WHO Label	Significant Clinical Characteristics	SIG Variant Classification*	Predominant Spike (S) Protein Mutations
A (19A)	Wuhan	One of the original haplotypes	VBM	D614G
B.1.427<br>B.1.429	Epsilon (Cal.20C)	Increased transmission; reduced susceptibility to antibodies	VBM	D614G, L452R, S13I, W152C
B.1.617.1<br>B.1.617.2	Delta (India)	Possible reduced susceptibility to antibodies	VOC	T19R, G142D, 156del, 157del, R158G, L452R, T478K, D614G, P681R, D950N
P.1	Gamma (Brazil)	Significantly reduced susceptibility to antibodies	VBM	E484K, K417T, N501Y
B.1.351	Beta (S. Africa)	Increased transmission; significantly reduced susceptibility to antibodies	VBM	L18F, D80A, D215G, 241-243del, K417N, E484K, N501Y, D614G, A701V
B.1.1.7	Alpha (U.K.)	Increased transmission; possible increased disease severity	VBM	69-70del, 144del, N501Y, A570D, D614G, P681H, T716I, S982A, D1118H

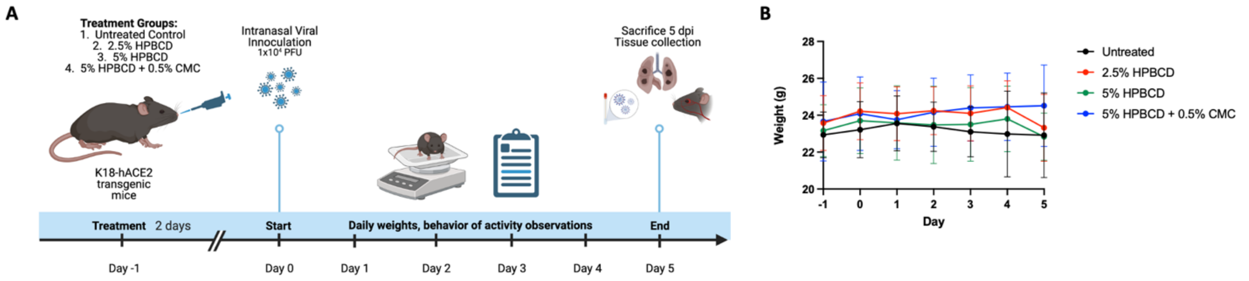

Supplementary Figure S1. K18-hACE2 mouse study design (A) 8 week-old K18-hACE2 SARS-CoV-2 study design using the SARS-CoV-2 Delta variant (B) Mouse weights recorded over the duration of study.
